# Survival analysis of electric vehicle charging behavior and the temporal evolution of feature effects

**DOI:** 10.1038/s41598-025-18771-8

**Published:** 2025-10-07

**Authors:** Matej Meža, Gregor Strle, Marko Meža

**Affiliations:** 1 Faculty of Electrical Engineering, University of Ljubljana, Ljubljana, Slovenia; 2 The Research Centre of the Slovenian Academy of Sciences and Arts (ZRC SAZU), Ljubljana, Slovenia

**Keywords:** Electric vehicle charging, Machine learning, Behavioral modeling, Churn prediction, Survival analysis, Energy infrastructure, Information technology, Psychology and behaviour, Energy and behaviour

## Abstract

This study proposes a survival-based modeling framework that combines behavioral features with interpretable machine learning to understand and predict user churn in electric vehicle charging services. Using a dataset of 1,074 users and 107,531 charging sessions from Central European countries, we modeled time-to-churn while handling censored observations. The best-performing model, a Stacked Weibull survival model based on gradient boosting, achieved a concordance index of 0.826 ± 0.041 and Integrated Brier Score of 0.078 ± 0.008 (5-fold cross-validation), with strong calibration relative to Kaplan-Meier survival estimates. Interpretability analyses identified sustained session frequency, positive engagement trends, and temporal regularity in charging behavior as key predictors of reduced churn risk. These findings highlight the potential of survival modeling integrated with behavioral analytics to predict churn risk and inform retention strategies in electric vehicle charging networks.

## Introduction

The transition to electric vehicles (EVs) depends critically on both vehicle availability and robust charging infrastructure. As charging networks expand globally, understanding user engagement patterns—particularly why and when users abandon charging services—has become important for operators, planners, and policymakers. This study focuses on predicting user churn, a phenomenon where customers disengage from charging services over time.

Churn prediction has been extensively studied in traditional sectors such as telecommunications, banking, and subscription services^1^. However, conventional classification approaches often overlook the temporal dynamics of disengagement, limiting their applicability to services like EV charging, where user engagement patterns are irregular and time-dependent^2,3^.

In the EV domain, prior research has examined charging behavior, segmented user populations by usage patterns^4,5^, and analyzed churn in public or shared charging networks^6,7^. While these studies offer valuable insights into user heterogeneity and service usage, they do not integrate survival modeling, behavioral feature engineering, and interpretability into a unified predictive framework.

Modeling churn in EV charging services presents distinct challenges. Charging session data are typically sparse, and session intervals vary considerably across users, complicating churn event identification. Furthermore, interpretability is essential to uncover the behavioral drivers of churn and to enable actionable interventions by service providers.

Successfully addressing these challenges can lead to accurate churn prediction, which in turn enhances service design and user retention strategies. By identifying behavioral patterns associated with disengagement, providers can target interventions more effectively–such as personalizing incentives or adapting infrastructure deployment–to reduce attrition and improve long-term service engagement.

To address these needs, this study introduces a survival-based modeling framework tailored to EV user churn prediction. The proposed approach combines detailed behavioral features derived from charging data, robust temporal cutoff strategies to avoid information leakage, and interpretable machine learning models. The framework estimates not only when users are likely to churn but also how specific behavioral dynamics contribute to that risk, enabling transparent and data-driven decision-making for EV service operators.

The remainder of this article is structured as follows. “Related work” reviews related work, “Materials and methods” details the materials and methods, “Results” presents the experimental results, and “Discussion” discusses the findings, outlines methodological considerations and limitations, and concludes with recommendations for future research.

## Related work

Churn modeling, defined as the prediction of customer attrition over time, has been widely studied in telecommunications, finance, and online services^1^. Traditional approaches often rely on classification models to predict whether a user will churn within a fixed window. Such models typically overlook the temporal structure of churn processes, treating churn as a static binary outcome rather than a time-to-event phenomenon.

Survival analysis provides a more appropriate framework for modeling time-varying churn. It estimates the probability of customer survival over time, accounts for right-censored observations, and models the hazard rate—the instantaneous risk of churn given survival up to that point. Despite its advantages, survival modeling remains underused in EV charging contexts.

An early contribution is Kim et al.^2^, who modeled inter-charge intervals using a heterogeneous hazard model. Their analysis of over 449,000 sessions from 9,027 primarily PHEV users in the Netherlands employed a latent class Cox model to capture behavioral segments such as regular and sporadic chargers. They incorporated time-varying covariates like weather and weekday effects to improve hazard estimates. The study focused on session-level timing rather than user-level survival, and its generalizability is limited given the focus on PHEV rather than pure EV users.

Understanding user heterogeneity has also motivated segmentation and clustering. Hu et al.^6^ used an entropy-weighted K-means algorithm to classify 7426 users based on a modified RFM framework, identifying high-value, potential, and lost users; such segmentation does not predict when churn occurs and overlooks censoring. Märtz et al.^7^ applied temporal clustering to 2.6 million German charging sessions, identifying usage profiles such as overnight home or daytime workplace charging, but did not address churn prediction or employ survival analysis. Closer to EV user segmentation with survival components, Pellegrini et al.^8^ combined a hazard-based model for inter-charging duration with latent classes to distinguish regular and irregular EV chargers; while informative about interval dynamics, this design does not model user-level churn or retention.

Survival analysis has also been applied in related mobility settings. Kostić et al.^9^ compared CoxPH with a deep survival model for car availability in free-floating EV carsharing and showed performance gains from modeling nonlinearities. In public transit, Yu et al.^10^ proposed a recurrent deep survival framework to predict lifecycle behavior status transitions from trip-chain sequences, demonstrating that survival methods can track evolving engagement beyond single churn events. At larger scale, Zhan et al.^11^ analyzed over 850 million EV driving and charging events from 1.6 million vehicles, revealing pronounced heterogeneity by vehicle type and time of day—patterns that may translate into different retention profiles even though churn itself was not modeled.

Other large-scale studies have examined charging behavior without focusing on churn. Helmus et al.^4^ used Gaussian mixture modeling on over 4.9 million Dutch sessions to identify 13 charging patterns and 9 user types. Weekx et al.^5^ analyzed habitual charging in Brussels, showing that routine users disproportionately affect network performance. Zhan et al.^11^ contrasted private and commercial fleets via spatio-temporal clustering. Several machine-learning–based studies have predicted charging behaviors. Chung et al.^12^ proposed an ensemble framework for session duration and energy use, combining SVR, Random Forests, and kernel density estimation. Shahriar et al.^13^ integrated weather and traffic into ensemble models, improving predictions. Ali et al.^14^ applied random-parameter hazard models to charging duration, identifying temporal and environmental predictors. Survival models using deep learning, such as DeepSurv^9^, have shown strong performance in other mobility contexts but have not been applied to EV charging churn prediction. These works focus on session-level outcomes rather than user-level churn.

Significant gaps remain. None of the studies reviewed combine detailed temporal behavioral features with user-level survival modeling. Approaches such as Hu et al.^6^, Märtz et al.^7^, and Pellegrini et al.^8^ segment users or model inter-charge intervals but do not estimate time to churn with censoring at the user level. Survival-based works such as Kim et al.^2^, Yassine et al.^3^, Kostić et al.^9^, and Yu et al.^10^ use temporal predictors yet rely on coarse or aggregated measures relative to the fine-grained behavioral dynamics we study. As noted by Shariatzadeh et al.^15^, multiple interacting behavioral factors—timing regularity, location diversity, and routine stability – shape charging behavior. Capturing these as temporal behavioral features is essential because they reflect individual routines and deviations that often precede churn, enabling earlier and more accurate risk estimation.

The present study addresses these limitations by integrating temporal behavioral features, survival modeling, temporal cutoff strategies, and interpretable machine learning tools. Compared to prior EV and related mobility studies^2,3,6–11^, which typically use aggregated or static predictors, our approach models detailed temporal dynamics of engagement, examines how feature effects vary over time via SHAP-based interpretation, and benchmarks classical and modern survival models under identical preprocessing while testing robustness to timeline confounding. This extends segmentation-oriented insights into a censoring-aware survival framework, improving prediction and interpretation of churn processes in EV charging services.

The next section describes the materials and methods used in this study.

## Materials and methods

This section presents the dataset, preprocessing steps, feature engineering and selection strategies, and the survival modeling approach employed in this study. The methodology is designed to leverage detailed behavioral data, ensure robustness against data leakage, and provide interpretable insights into the factors driving user churn in EV charging services.

### Dataset overview and preprocessing

The analysis utilized an anonymized EV charging dataset comprising 13,056 users and 159,624 sessions recorded between March 2021 and December 2024 across Slovenia and neighboring countries (Austria, Italy, Hungary, and Croatia). The dataset, provided by Megatel d.o.o., included user registration dates, session timestamps, durations, energy (Wh), locations, and payment types. Informed consent was gathered and personally identifiable data, such as user IDs and geographic coordinates, were anonymized at the source.

To ensure sufficient behavioral history and reduce noise from sparsely observed users, we required a minimum of 30 sessions for inclusion in modeling. This choice was guided by a stability analysis (Supplementary Fig. S4) comparing thresholds from 10 to 40 sessions. Higher thresholds yielded longer average service durations and modestly higher monthly session rates, but substantial inter-session gaps persisted even at the strictest threshold. Importantly, Kaplan-Meier survival analysis showed that users with $\ge 30$ sessions had markedly higher survival probability (median not reached, indicating that more than half of these users remained active at the end of the observation period) compared to those with $<30$ sessions (median = 4 days; log-rank $\chi ^2=1651.2$, $p<0.001$; Cox proportional hazards model hazard ratio = 0.138, 95% CI [0.123, 0.154]). The $\ge 30$ group also exhibited greater stability in survival curves, indicating reduced sensitivity to random early dropouts and mitigating potential bias from including very low-activity users. This threshold therefore balances representativeness, behavioral stability, and statistical power. The final modeling sample comprised 1,074 users (8.2%) and 107,531 sessions (67.4%).

Charging activity followed a weekly rhythm, peaking on Fridays (16.8%) and dipping on Sundays (8.8%), consistent with commuting-related charging patterns. Session volumes rose notably after mid-2022, reflecting growing EV adoption and post-pandemic recovery. Inter-session gaps were highly skewed (Supplementary Fig. S1): the median gap was 1.0 day, the 75th percentile 5.0 days, and the 90th percentile 13.0 days, indicating that occasional gaps of two or more weeks are normal even among engaged users. These statistics informed the selection of a conservative inactivity window for churn labeling.

### Churn definition and cutoff strategy

Churn was modeled as a survival prediction task, addressing right-censored observations and irregular event times. A single global reference date was set to the 75th percentile of users’ last recorded session dates (December 13, 2024; Supplementary Fig. S2) to reduce endpoint bias by avoiding extreme early or late users. This date falls immediately before the Christmas and New Year holiday period, when many users temporarily reduce charging.

Using a fixed reference date standardizes censoring across the cohort. Without it, users who joined shortly before the analysis date could not have accumulated the inactivity window and would therefore be systematically labeled as right-censored, inflating survival estimates for recent joiners. Applying the same cutoff to all users ensures that these recent joiners are uniformly right-censored at the same time point, preventing such bias.

Users whose last session occurred more than 60 days before the reference date were labeled as churned ($\textrm{event}=1$), while those with a last session within the 60-day window were labeled as right-censored ($\textrm{event}=0$). Feature extraction was performed using only sessions that occurred before the cutoff date $(\text {reference date} - 60 \text { days})$, ensuring strict temporal separation between predictor data and outcome labeling to prevent information leakage.

The inactivity threshold of 60 days was selected based on empirical evidence and seasonal considerations. First, the empirical gap distribution (Supplementary Fig. S1) shows that the 90th percentile inter-session gap is 13 days—less than one-quarter of the threshold—making this window conservative for detecting disengagement. Second, seasonal effects, such as holiday-related usage reductions, were explicitly considered when defining the churn strategy, as shorter inactivity windows may misclassify temporarily inactive but still engaged users. Extended inactivity can occur during major holiday or travel periods beyond the Christmas/New Year season visible in our data (Supplementary Fig. S2). Third, window-size sensitivity analysis (Supplementary Fig. S3) shows that churn prevalence decreases from about 40% at 30 days to about 32% at 60 days, with minimal change beyond 90 days.

### Feature engineering

Features were designed to capture usage intensity, behavioral dynamics, and routine patterns. They were grouped into: (i) basic usage features, (ii) dynamic features (e.g., trends and volatility), and (iii) routine features (e.g., regularity and location habits). Table 1 defines the full set used in modeling. All features were standardized (z-score) using scikit-learn’s StandardScaler to ensure comparability and numerical stability.

**Table 1:** Summary of behavioral features used in churn modeling, grouped into basic, dynamic, and routine categories.

Category	Feature	Definition
Basic	avg_energy_wh	Average energy consumed per session
Basic	days_active	Number of unique active days
Basic	first_session_days_ago	Days since first recorded session
Basic	last_session_days_ago	Days since last recorded session
Basic	std_energy_wh	Standard deviation of energy consumed
Basic	total_energy_wh	Total energy consumed
Basic	total_sessions	Total number of charging sessions
Basic	unique_locations	Number of unique locations visited
Behavioral (dynamic)	trend_energy	Slope of energy consumption over time
Behavioral (dynamic)	trend_session_weekly	Slope of weekly session count over time
Behavioral (dynamic)	energy_velocity	Rate of change in energy consumption
Behavioral (dynamic)	energy_acceleration	Change in rate of energy consumption (2nd derivative)
Behavioral (dynamic)	cumulative_energy	Total energy consumed across all sessions
Behavioral (dynamic)	energy_latency_days	Days between last session and reference date
Behavioral (dynamic)	peak_energy_count	Number of significant peaks in energy consumption
Behavioral (dynamic)	peak_energy_width	Average width of energy consumption peaks
Behavioral (dynamic)	peak_energy_prominence	Average prominence of energy consumption peaks
Behavioral (dynamic)	peak_session_freq_count	Number of peaks in weekly session frequency (computed over fixed-length windows)
Behavioral (dynamic)	rolling_energy_mean	7-day rolling average of energy consumption
Behavioral (dynamic)	rolling_energy_std	Standard deviation of 7-day rolling energy
Behavioral (dynamic)	location_entropy	Diversity of location usage
Behavioral (dynamic)	location_switch_rate	Rate at which user changes charging locations
Behavioral (dynamic)	peak_location_change_count	Number of location switching spikes
Behavioral (routine)	weekday_pattern_index	Consistency in day-of-week charging patterns (lower = more consistent)
Behavioral (routine)	timeblock_entropy	Entropy of charging distribution across time blocks
Behavioral (routine)	session_interval_regularity	Consistency in time between sessions
Behavioral (routine)	primary_location_pct	Percentage of sessions at most frequent location
Behavioral (routine)	location_exploration_rate	Rate of discovering new locations
Behavioral (routine)	short_session_pct	Percentage of short sessions
Behavioral (routine)	long_session_pct	Percentage of long sessions
Behavioral (routine)	behavioral_consistency_score	Regularity in session timing, energy, and location
Behavioral (routine)	disruption_frequency	Count of interruptions in routine behavior
Behavioral (routine)	avg_disruption_length_days	Average length of disruptions in days

### Feature selection

Feature selection followed a multi-stage process. First, features with zero variance or over 20% missingness were removed. Remaining missing values were imputed via linear interpolation over session timelines, falling back to mean imputation. Second, features with Variance Inflation Factor (VIF) above 10.0 were excluded to reduce multicollinearity. Third, Recursive Feature Elimination (RFE) with an XGBoost regressor trained on log-transformed durations ranked features by predictive value. Finally, features highly correlated with time-to-churn or churn status (Spearman’s $\rho > 0.6$) were excluded to avoid label leakage. This pipeline ensured a predictive, interpretable, and leakage-free feature set.

### Survival modeling approach

Three XGBSE-based survival models were evaluated: KaplanNeighbors (KN), Stacked Weibull (SW), and Debiased Binary Cross-Entropy (DBCE)^16^. In addition, two classical approaches were included for comparison: the Cox Proportional Hazards (CoxPH) model^17^ and the Random Survival Forest (RSF)^18^. The CoxPH model provides a semi-parametric reference with a multiplicative hazard structure, while RSF offers a non-parametric tree-ensemble alternative capable of capturing complex, non-linear feature interactions.

All models were trained and evaluated using the same feature set obtained from the preprocessing and feature selection pipeline, ensuring that differences in performance reflect modeling capacity rather than differences in input variables.

Model performance was assessed using 5-fold cross-validation with two primary metrics: the concordance index (C-index), measuring discrimination ability by ranking survival times correctly (higher is better), and the Integrated Brier Score (IBS), measuring calibration through predicted-actual probability differences over time (lower is better). The best model underwent nested 5-fold cross-validation for unbiased generalization estimates, with the outer loop assessing performance and the inner loop applying feature selection and model fitting. No hyperparameter optimization was performed to isolate the effects of feature engineering and model choice.

Final models were retrained on the full dataset using the features selected through a combination of multicollinearity reduction (via VIF), gain-based importance filtering, and post-selection correlation checks.

Durations were converted from days to months to aid interpretability. Evaluation was conducted at the 25th, 50th, and 75th percentiles of observed durations. Feature effects on time-to-churn for the XGBSE models were analyzed using Partial Dependence Plots (PDPs) and Individual Conditional Expectation (ICE) curves.

Prediction uncertainty for the XGBSE models was quantified using bootstrap resampling with 100 replicates, producing 90% confidence intervals for survival curves. SHAP (SHapley Additive exPlanations) values were computed using a mirror XGBoost regressor trained on the same input to interpret feature contributions. SHAP summary plots and dependency plots revealed key behavioral drivers of churn risk.

Temporal variation in feature importance was examined by tracking SHAP values across time. Additionally, SHAP-weighted density plots were used to highlight systematic behavioral differences between churned and censored users.

### Software and libraries

All data processing, feature engineering, modeling, and interpretability analyses were conducted using Python v3.10. Key libraries included scikit-learn (v1.6.1) for preprocessing and machine learning utilities^19^, XGBoost (v2.1.1) for gradient boosting models^20^, and XGBSE (v0.3.3) for survival modeling with boosted trees^16^. Survival analysis was further supported by lifelines (v0.29.0)^21^. Model interpretation was carried out using the SHAP library (v0.46.0) for Shapley value computation^22^. Visualization and statistical analyses were performed using seaborn (v0.13.2)^23^ and scipy (v1.13.1)^24^.

### Pipeline overview

**Fig. 1:**
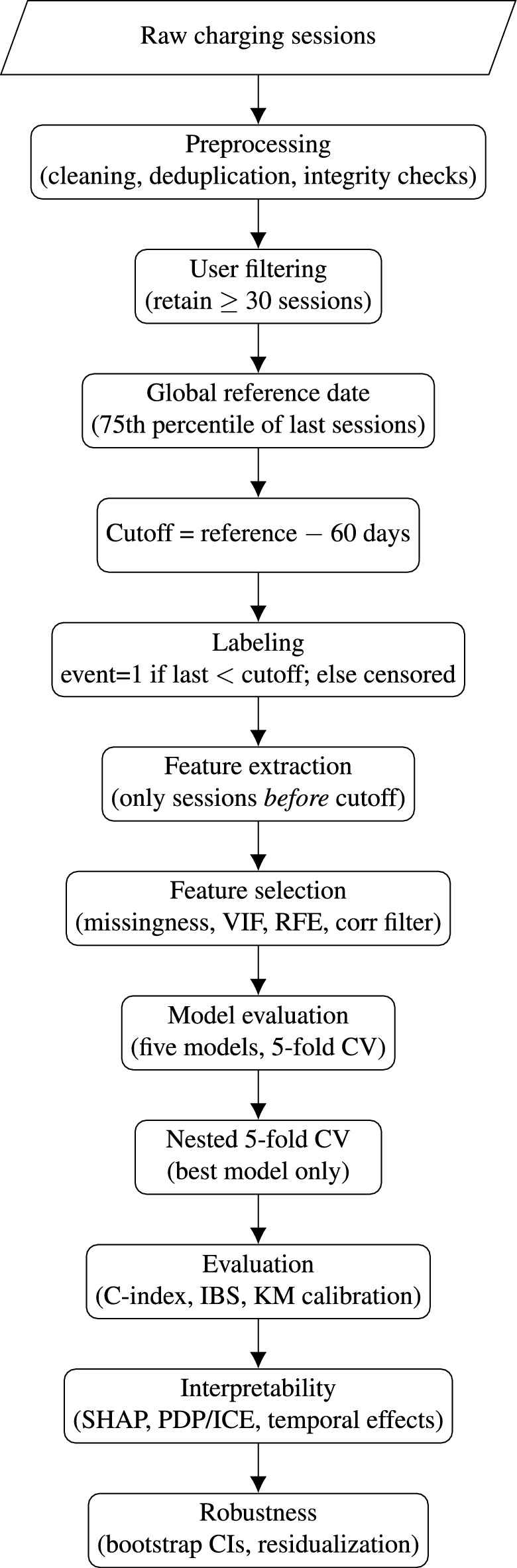
End-to-end pipeline. After preprocessing and filtering to retain users with at least 30 sessions, a global reference date (75th percentile of last sessions) and a 60-day look-back define churn labels. Features are computed from sessions prior to the cutoff, selected via missingness, multicollinearity, and importance criteria. All five models are evaluated using 5-fold cross-validation; the best model is further assessed with nested 5-fold CV for unbiased generalization. Interpretability and robustness analyses complete the workflow.

Figure 1 provides a visual summary of the end-to-end workflow, from raw data preprocessing and user filtering to churn labeling, feature engineering, survival modeling, evaluation, and interpretability analyses. The diagram complements the described in “Materials and methods”, making the sequence of steps and their dependencies explicit.

## Results

This section presents the key findings of the study, starting with the results of the feature selection process and the performance evaluation of the survival models. The analyses then delve into the interpretation of feature effects on user churn risk, including global and individual-level patterns, temporal evolution of feature importance, and notable feature interactions. The results collectively provide insights into the behavioral factors driving user retention and disengagement in the EV charging service context.

### Feature importance

The final feature set was obtained through a combination of multicollinearity filtering, correlation analysis with survival targets, and gain-based feature selection. This resulted in 14 behavioral features that capture diverse temporal and spatial aspects of user engagement.

To support interpretability, features were ranked using mean absolute SHAP values derived from a mirror XGBoost regressor. Table 2 lists the selected features in order of predictive contribution. The top-ranked variables—peak_session_freq_count, std_weekly_sessions, session_interval_regularity, and session_freq_trend—indicate the importance of usage consistency in predicting churn risk.

**Table 2:** Final set of selected features ranked by mean absolute SHAP value, with their respective categories from Table 1. These 14 behavioral features were used in all survival models for a default 60-days churn window.

Feature name	Category	SHAP importance
peak_session_freq_count	Behavioral (dynamic)	0.219
std_weekly_sessions	Behavioral (dynamic)	0.170
session_interval_regularity	Behavioral (routine)	0.160
session_freq_trend	Behavioral (dynamic)	0.151
location_switch_rate	Behavioral (dynamic)	0.129
weekday_pattern_index	Behavioral (routine)	0.126
location_exploration_rate	Behavioral (routine)	0.102
energy_acceleration	Behavioral (dynamic)	0.097
energy_consumption_trend	Behavioral (dynamic)	0.095
session_duration_trend	Behavioral (dynamic)	0.091
peak_energy_count	Behavioral (dynamic)	0.074
energy_velocity	Behavioral (dynamic)	0.067
timeblock_entropy	Behavioral (routine)	0.057
peak_location_change_count	Behavioral (dynamic)	0.049

### Model performance

Five survival models were evaluated under identical conditions: Cox Proportional Hazards (CoxPH), Random Survival Forests (RSF), and three XGBSE variants (KaplanNeighbors, StackedWeibull, DebiasedBCE). Performance was assessed using 5-fold cross-validation on the final feature set.

The top-ranked feature peak_session_freq_count showed correlation with user duration (Spearman’s $\rho \approx 0.60$). Since this feature accumulates over weekly bins, it captures both behavioral intensity and observation time. To isolate behavioral effects from timeline correlation, we residualized the feature by removing its duration-dependent component through regression. Table 3 reports performance using both original and residualized features across three churn windows (30, 60, and 90 days), with 60 days as default.

**Table 3:** Cross-validated performance (5-fold) of the models on the final feature set for three churn window definitions (30, 60 (default), and 90 days), comparing the original peak_session_freq_count feature with its residualized variant to test robustness against timeline confounding. For each churn window and feature setting (original vs. residualized), the highest C-index and lowest IBS are bolded separately to highlight trade-offs between discrimination and calibration.

Window	Model	Original feature		Residualized feature	
(days)		C-index	IBS	C-index	IBS
30	CoxPH	$\mathbf {0.836} \pm 0.023$	$0.103 \pm 0.007$	$0.738 \pm 0.032$	$0.126 \pm 0.010$
	RSF	$0.807 \pm 0.015$	$0.097 \pm 0.008$	$0.767 \pm 0.012$	$0.108 \pm 0.010$
	XGBSE KN	$0.822 \pm 0.032$	$\mathbf {0.078} \pm 0.005$	$\mathbf {0.781} \pm 0.025$	$\mathbf {0.086} \pm 0.008$
	XGBSE SW	$0.816 \pm 0.033$	$0.086 \pm 0.008$	$0.777 \pm 0.031$	$0.094 \pm 0.006$
	XGBSE DBCE	$0.680 \pm 0.047$	$0.138 \pm 0.013$	$0.537 \pm 0.009$	$0.168 \pm 0.010$
60	CoxPH	$\mathbf {0.830} \pm 0.026$	$0.106 \pm 0.007$	$0.718 \pm 0.039$	$0.128 \pm 0.007$
	RSF	$0.820 \pm 0.019$	$0.094 \pm 0.008$	$0.782 \pm 0.021$	$0.104 \pm 0.008$
	XGBSE KN	$0.811 \pm 0.047$	$0.079 \pm 0.008$	$0.777 \pm 0.039$	$0.087 \pm 0.006$
	XGBSE SW	$0.826 \pm 0.041$	$\mathbf {0.078} \pm 0.008$	$\mathbf {0.795} \pm 0.031$	$\mathbf {0.086} \pm 0.008$
	XGBSE DBCE	$0.711 \pm 0.053$	$0.125 \pm 0.014$	$0.542 \pm 0.006$	$0.158 \pm 0.010$
90	CoxPH	$0.804 \pm 0.036$	$0.107 \pm 0.003$	$0.656 \pm 0.044$	$0.128 \pm 0.005$
	RSF	$0.793 \pm 0.042$	$0.092 \pm 0.008$	$\mathbf {0.703} \pm 0.039$	$0.109 \pm 0.009$
	XGBSE KN	$0.818 \pm 0.025$	$0.078 \pm 0.009$	$0.697 \pm 0.035$	$\mathbf {0.099} \pm 0.009$
	XGBSE SW	$\mathbf {0.830} \pm 0.030$	$\mathbf {0.076} \pm 0.009$	$0.689 \pm 0.023$	$0.114 \pm 0.009$
	XGBSE DBCE	$0.688 \pm 0.049$	$0.124 \pm 0.011$	$0.498 \pm 0.006$	$0.158 \pm 0.011$

With original features, CoxPH achieved highest discrimination (C-index $=0.830 \pm 0.026$) but poorest calibration (IBS $=0.106 \pm 0.007$), indicating good ranking ability but systematic probability miscalibration. RSF showed slightly lower discrimination ($0.820 \pm 0.019$) with improved calibration ($0.094 \pm 0.008$). Among XGBSE models, KaplanNeighbors achieved low IBS ($0.079 \pm 0.008$) at the cost of discrimination ($0.811 \pm 0.047$). StackedWeibull balanced both metrics effectively, achieving strong discrimination ($0.826 \pm 0.041$) with the lowest IBS ($0.078 \pm 0.008$)–a gap of 0.028 compared to CoxPH, exceeding three times the standard error. Since survival model selection requires balancing discrimination and calibration, StackedWeibull emerged as the best overall performer.

Under residualization, discrimination decreased across all models, most dramatically for CoxPH ($0.830 \rightarrow 0.718$), while calibration changes remained modest. StackedWeibull maintained best joint performance (C-index $=0.795 \pm 0.031$, IBS $=0.086 \pm 0.008$) with the smallest relative discrimination drop among top models, suggesting it captures behavioral patterns beyond timeline artifacts.

The XGBSE DebiasedBCE variant performed substantially worse than all other models across all conditions. With original features at 60 days, its C-index ($0.711 \pm 0.053$) and IBS ($0.125 \pm 0.014$) indicated both poor discrimination and calibration. After residualization, performance degraded to near-random levels (C-index $\approx 0.54$). This consistent underperformance across all churn windows suggests convergence issues with this dataset.

Based on its balanced performance in both settings, XGBSE StackedWeibull was selected for subsequent analyses. Nested 5-fold cross-validation confirmed generalization ability (C-index $=0.824$, IBS $=0.083$), closely matching standard CV metrics.

Alternative churn windows revealed expected trade-offs. The 30-day window yielded marginally higher discrimination but increased false positive risk from temporary inactivity. The 90-day window reduced discrimination across all models while maintaining StackedWeibull’s relative advantage. Consistent with Section 4.2, the 60-day window provided optimal balance between sensitivity and specificity while avoiding misclassification of seasonal patterns.

### Survival calibration, model uncertainty, and discrimination ability

**Fig. 2:**
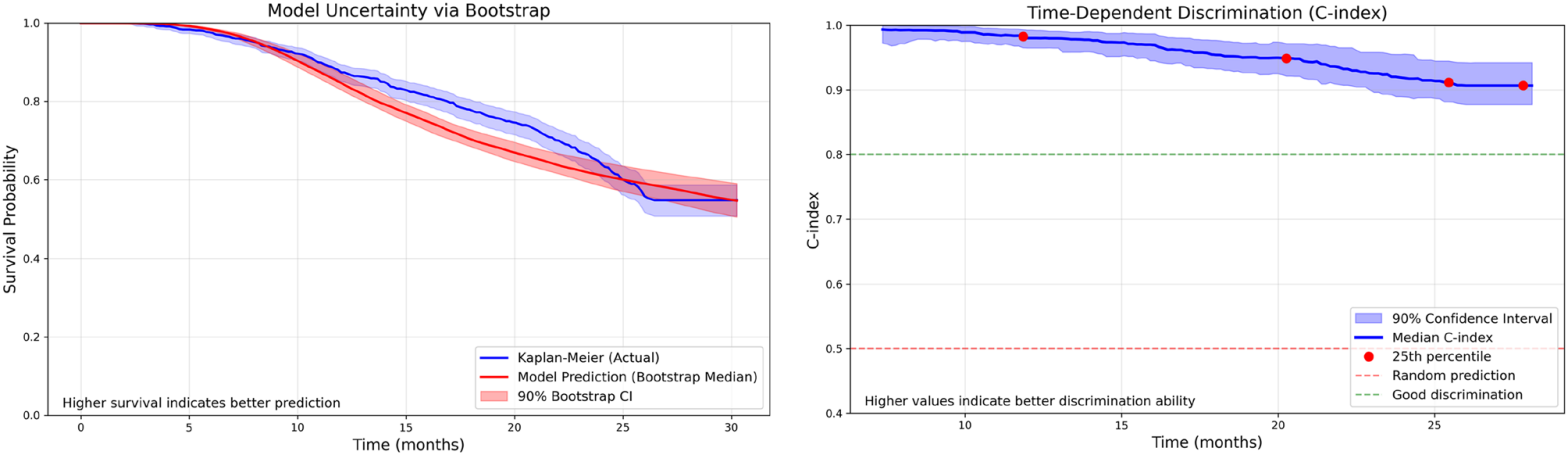
Model calibration and discrimination evaluation for the StackedWeibull model. Left: comparison between model-predicted median survival and Kaplan-Meier empirical survival estimate, with shaded regions representing 90% bootstrap-derived confidence intervals. Right: time-dependent concordance index (C-index) evaluating risk stratification quality across the timeline. Both calibration and discriminative ability remain strong throughout the observation window.

As shown in Fig. 2, the StackedWeibull model demonstrated strong calibration across the observation window. Calibration was evaluated by comparing the predicted survival curve to the empirical Kaplan-Meier estimate, which summarizes observed survival probabilities without assuming any parametric form.

The model’s median survival predictions closely followed the Kaplan-Meier curve and remained within the 90% bootstrap confidence intervals over most of the observation period. Slight deviations and widening intervals were observed at later horizons due to increased censoring.

Model uncertainty, visualized via bootstrap confidence bands, was minimal during the early and middle time frames and grew only moderately after 25 months, reflecting fewer observed churn events.

The right panel of Fig. 2 shows the time-dependent C-index, measuring discriminative ability over time. Discrimination was strong in the earliest periods (C-index $\approx 0.98$) when early churn events were easier to rank. As time progressed and events occurred across a wider range of predicted risks, the C-index gradually declined to $\approx 0.90$ by 28.8 months. Confidence intervals remained narrow throughout, indicating consistent separation between users with different survival prospects.

Together, these results demonstrate that the StackedWeibull model maintained strong calibration, low uncertainty, and reliable discrimination throughout the observation window.

### Global feature effects

**Fig. 3:**
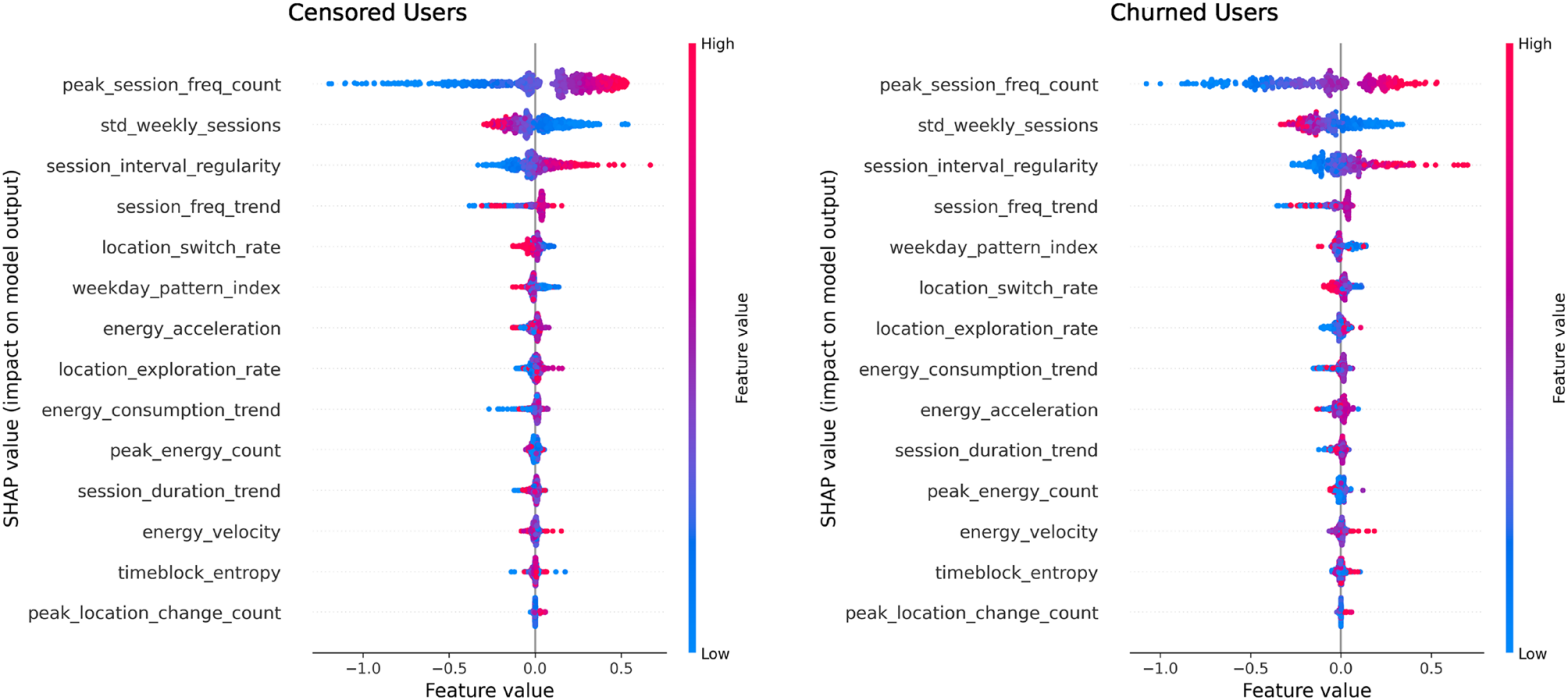
Global SHAP summary plots for survival predictions. Left: churned users, where low peak session frequency and negative session growth rates significantly contribute to churn risk. Right: censored users, where stability in session frequency and location behavior supports longer user survival.

Global SHAP analysis confirmed that peak_session_freq_count and session_freq_trend were the dominant contributors to churn risk predictions across both churned and censored user populations. Low session intensity and declining engagement trends were associated with elevated churn risk among churned users. For censored users, consistent session frequency, stable weekday patterns, and predictable location usage were associated with lower churn risk (Fig. 3).

A corresponding SHAP summary for the residualized model is provided in Supplementary Fig. S7, showing similar feature rankings and effect directions. These results reinforce the behavioral relevance and robustness of the original predictors.

#### SHAP intensity vs. survival risk alignment

**Fig. 4:**
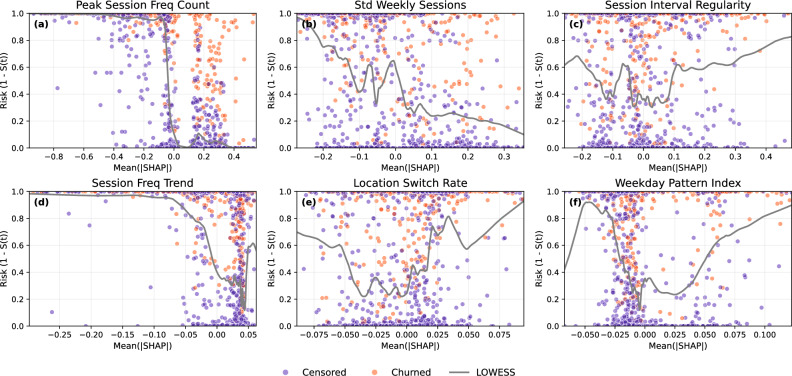
SHAP intensity versus predicted churn risk across users for selected behavioral features. (**a**–**f**) Per-user scatterplots of mean absolute SHAP values against predicted churn risk (1 - *S*(*t*)). Grey LOWESS trend lines capture the non-linear relationship between each feature’s SHAP intensity and survival risk. Users are colored by event status (blue = censored, red = churned).

To evaluate how feature-level SHAP intensity aligns with predicted churn risk, we visualized the relationship between each user’s mean absolute SHAP value and their predicted risk score (1 - *S*(*t*)). Figure 4 presents scatterplots for six top-ranked features, with users colored by churn status and smoothed LOWESS trend lines shown in grey.

Several key patterns emerge. For peak_session_freq_count and session_freq_trend, higher SHAP intensity is positively aligned with higher predicted churn risk, especially among churned users—indicating that the model heavily relies on these features to flag high-risk individuals. In contrast, features such as weekday_pattern_index show flatter trends, suggesting a weaker or less targeted contribution to risk estimation.

These results confirm that global SHAP attributions correspond well with individualized risk scores. A residualized version of this analysis (Supplementary Fig. S6) shows consistent alignment patterns.

### Feature effects across time horizons

**Fig. 5:**
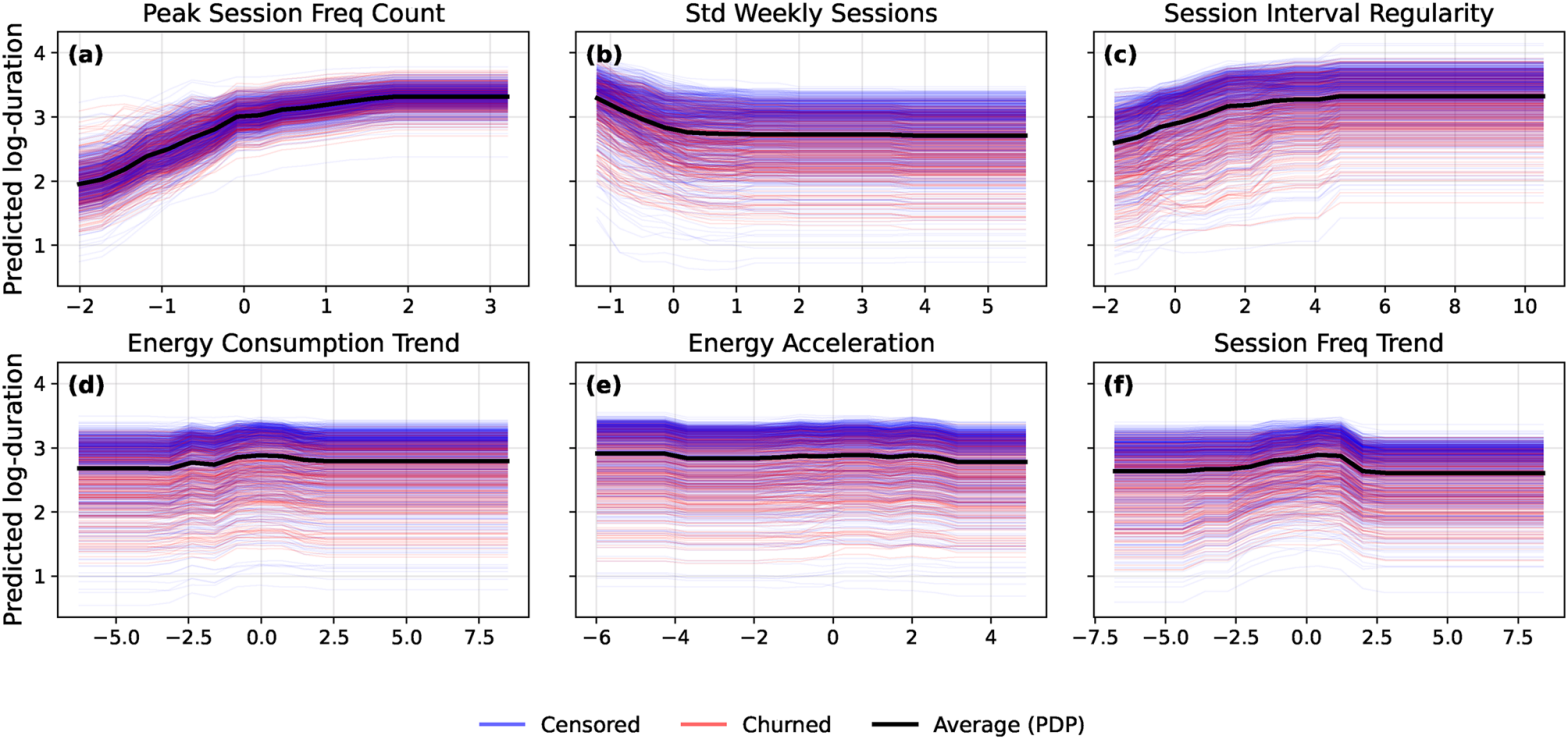
Partial dependence and ICE plots for selected behavioral features: (**a**) peak_session_freq_count, (**b**) std_weekly_sessions, (**c**) session_interval_regularity, (**d**) energy_consumption_trend, (**e**) energy_acceleration, (**f**) session_freq_trend. Higher engagement stability, regularity, and positive energy behaviors are generally associated with improved survival probabilities. Individual user trajectories are colored by churn status (churned vs. censored).

Partial dependence (PDP) and Individual Conditional Expectation (ICE) analyses reveal how key behavioral features influence survival outcomes over time. Figure 5 presents PDP and ICE curves for six top-ranked features, capturing nonlinear effects and user-level heterogeneity.

Several consistent patterns emerge. peak_session_freq_count (plot a) exhibits a strong monotonic relationship, with higher values linked to longer survival durations – indicating that sustained high engagement reduces churn risk. std_weekly_sessions (plot b) shows a U-shaped effect, where both low and high variability increase churn probability, suggesting that moderate consistency is associated with longer survival. session_interval_regularity (plot c) demonstrates a plateauing benefit, where increased regularity improves survival up to a threshold.

Energy-related features, including energy_consumption_trend (plot d) and energy_acceleration (plot e), show modest positive associations, implying that increasing or stable energy usage patterns are associated with longer survival times. Finally, session_freq_trend (plot f) confirms that growing engagement over time predicts reduced churn risk.

Supplementary Figs. S5–S7 present corresponding PDP/ICE plots using residualized feature variants, confirming the robustness of these patterns.

#### Temporal feature evolution

**Fig. 6:**
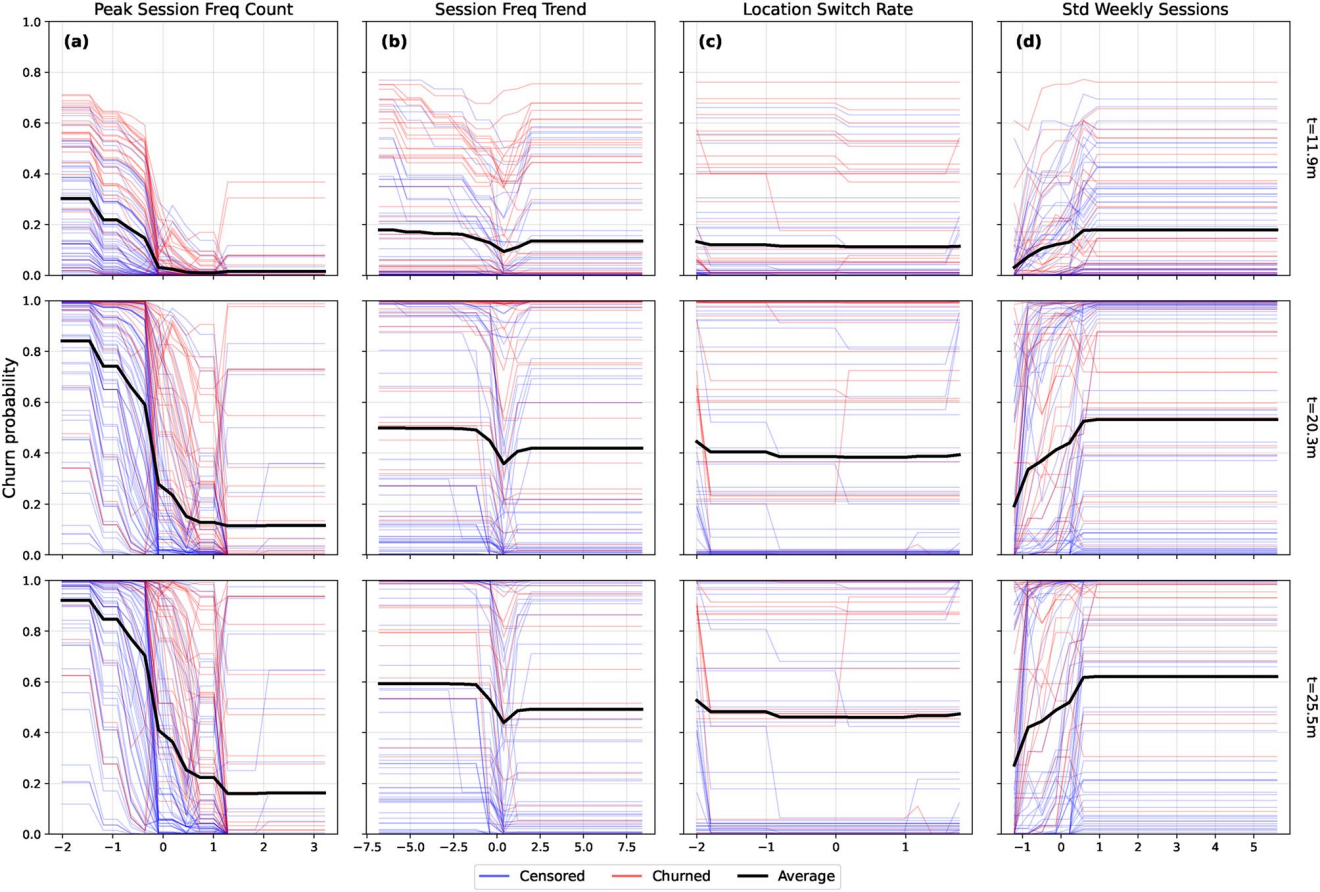
Temporal evolution of churn probability across key behavioral features at different time horizons: (**a**) peak_session_freq_count, (**b**) session_freq_trend, (**c**) location_switch_rate, (**d**) std_weekly_sessions. Stable and growing engagement patterns consistently correlate with lower churn risk across time, while users with low or deteriorating engagement behaviors experience increased risk as time progresses.

Beyond marginal effects, the temporal dynamics of behavioral predictors were examined using a feature evolution grid (Fig. 6), which visualizes predicted churn probability across standardized feature values at three representative time horizons (11.9, 20.3, and 25.5 months).

Churn risk remained consistently lower for users with high peak_session_freq_count across all time points, with increasing separation between churned and censored users over time. Positive session_freq_trend values consistently predicted lower churn probability, highlighting the importance of sustained or increasing usage. Location_switch_rate indicated that moderate switching among familiar locations was associated with lower risk, whereas low switching elevated risk, particularly at longer durations. std_weekly_sessions reinforced the previously observed U-shaped pattern: both highly erratic and overly uniform weekly behaviors were linked to elevated risk, with the distinction sharpening over time.

A complete time-stratified analysis of all 14 features is provided in Supplementary Fig. S8.

#### Feature interactions

**Fig. 7:**
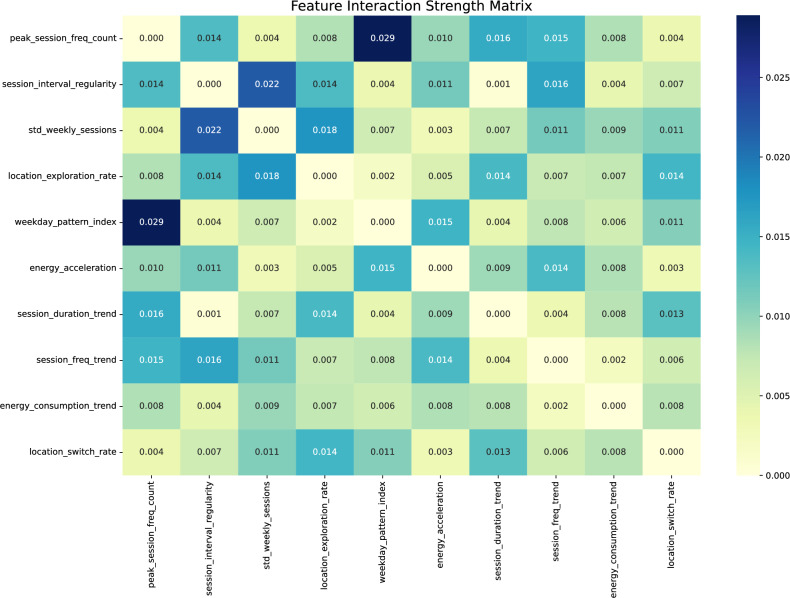
Feature interaction matrix based on SHAP interaction values. Strongest interactions appear between peak_session_freq_count and weekday_pattern_index (0.032), and among location-based features. Darker cells indicate stronger average pairwise interaction importance.

Interaction analysis (Fig. 7) revealed complex synergistic effects between behavioral features. The interaction matrix identifies several key relationships that amplify or modulate churn risk beyond individual feature contributions.

The strongest interaction emerged between peak_session_freq_count and weekday_pattern_index (interaction strength = 0.032), suggesting that session frequency peaks combine with weekday consistency to create a compound effect on survival probability. Users maintaining both high session peaks and regular weekday patterns showed disproportionately lower churn risk compared to either feature alone. This multiplicative relationship indicates that sustained intensity requires temporal regularity to minimize churn risk.

Critically, the interaction between peak_session_freq_count and session_freq_trend reveals that concurrent low values in both features sharply increase churn risk, underscoring the combined importance of session intensity and positive engagement momentum. The interaction with session_duration_trend (0.018) further suggests that high-frequency users who also increase session duration over time exhibit particularly low churn probability—a pattern consistent with deepening service integration.

Notable secondary interactions include std_weekly_sessions with session_interval_regularity (0.020), where the combination of moderate weekly variation with consistent inter-session timing characterizes users with longer survival times.

Location-based features showed rich interdependencies. The location_switch_rate and location_exploration_rate interaction (0.017) revealed distinct user archetypes: users with high switching among familiar locations (high switch, low exploration) demonstrated lower churn risk, indicating habitual multi-location usage patterns. Conversely, high exploration with irregular switching suggested unstable usage contexts associated with elevated churn risk (Supplementary Fig. S9). The location_exploration_rate interaction with session_duration_trend (0.015) further indicates that users exploring new locations while maintaining consistent session durations show longer survival times than those with erratic patterns across both dimensions.

Energy-related interactions, while weaker, revealed meaningful patterns. The energy_acceleration and weekday_pattern_index interaction (0.014) suggests that users with stable weekday patterns can better accommodate energy usage changes, while those with irregular patterns show increased churn risk when energy consumption fluctuates.

These interaction patterns suggest that churn prediction requires considering feature combinations rather than isolated behaviors. The synergistic effects between temporal regularity, spatial stability, and usage intensity create a multidimensional landscape where behavioral consistency across multiple dimensions provides cumulative protection against churn.

## Discussion

The survival-based modeling framework presented in Section 5 achieved a cross-validated concordance index (C-index) of $0.826 \pm 0.041$ and integrated Brier Score (IBS) of $0.078 \pm 0.008$ using a final set of 14 behavioral features, outperforming traditional Cox models (C-index $0.830 \pm 0.026$, IBS $0.106 \pm 0.007$) and Random Survival Forests (C-index $0.820 \pm 0.019$, IBS $0.094 \pm 0.008$) in balancing discrimination and calibration. These features, derived from multicollinearity filtering, survival correlation analysis, and gain-based ranking, captured complementary temporal and spatial aspects of engagement. The top-ranked predictors – peak_session_freq_count (SHAP importance 0.219), std_weekly_sessions (0.170), session_interval_regularity (0.160), and session_freq_trend (0.151)—consistently contributed most to churn risk predictions.

Robustness checks using a residualized peak_session_freq_count warrant further discussion. Residualization removes not only the duration-correlated component but also legitimate behavioral patterns that develop over time. The differential impact across models provides evidence that behavioral effects dominate: StackedWeibull’s modest decrease (3.8%) contrasted sharply with CoxPH’s substantial degradation (13.5%), suggesting that tree-based models capture behavioral patterns beyond linear duration dependencies. Moreover, StackedWeibull’s post-residualization C-index of 0.795 far exceeds random prediction (0.5), with maintained calibration (IBS increasing only from 0.078 to 0.086). These results demonstrate that while peak_session_freq_count contains duration information, its behavioral component provides the primary predictive value. The residualized version represents a conservative lower bound of model performance after removing both timeline effects and time-evolved behavioral patterns.

Model calibration closely tracked Kaplan-Meier estimates throughout the 30-month window, with only moderate widening of uncertainty intervals at later horizons due to censoring. As shown in Fig. 2, the time-dependent C-index was high in early periods and declined gradually to approximately 0.87 by 28.8 months, consistent with the overall cross-validated value and reflecting the increasing difficulty of churn risk discrimination at longer horizons.

### Behavioral interpretation and relation to prior work

SHAP-based interpretation revealed that declining engagement momentum and reduced charging regularity were strong indicators of churn risk. Users with irregular weekly session patterns or negative session frequency trends had substantially higher hazard rates. These findings are consistent with Kim et al.^2^, who identified high-frequency, regular chargers as having lower hazards, and with Hu et al.^6^, who found that frequent, stable users sustain service use. Weekx et al.^5^ similarly demonstrated that habitual users disproportionately contribute to network utilization. Our results extend these observations by showing that temporal changes in regularity—not just static measures—predict churn, and that early declines in frequency or energy use are particularly informative.

Spatial behavior also played a key role. Users who switched between stations often but within a limited set showed longer survival times. This complements Yassine et al.^3^, who showed that stable station use was associated with longer membership in a carsharing service, linking this pattern to broader vehicle availability effects in shared mobility. Our results suggest that such spatial stability may reduce churn risk even when multiple locations are used. In contrast, high location exploration without stable switching was associated with higher churn risk, potentially reflecting service dissatisfaction or unstable charging routines.

Interaction effects, especially between location_switch_rate and location_exploration_rate (Supplementary Fig. S9), reinforce the need to examine behavioral features jointly. This aligns with Shariatzadeh et al.^15^, who found that timing regularity, location diversity, and routine stability interact to shape charging behavior. We find that certain combinations – for example, high exploration with sporadic switching—can signal elevated churn risk, whereas habitual patterns in both time and space tend to reduce churn probability.

While our findings demonstrate consistent associations between temporal regularity, spatial stability, and churn risk, their generalizability across regions and user segments warrants further investigation. Studies from the Netherlands^2,4^, Germany^7,8^, China^10,11^, and the United States^3^ confirm that behavioral routines and infrastructure coverage shape engagement, though specific patterns vary with policy incentives, urban form, and fleet ownership. For instance, Zhan et al.^11^ found divergent patterns between private and commercial fleets in China, while Pellegrini et al.^8^ identified regular versus irregular charging behaviors among German BEV users, closely aligning with our regularity—churn link. Recent reviews^15^ further emphasize interaction effects between temporal behavior and infrastructure availability.

Compared to other survival-based mobility studies, such as Kostić et al.^9^ on EV carsharing and Yu et al.^10^ on transit lifecycle transitions, our approach uses more granular temporal features and models the user-level churn event directly. This granularity allows early identification of behavioral deviations that may precede disengagement. Overall, evidence across temporal, spatial, and interaction effects supports the conclusion that sustained, regular behavior in both time and location is associated with lower churn risk, while volatility is an early warning signal for churn.

### Methodological considerations

All models were evaluated under identical preprocessing, feature selection, and validation conditions (Table 3) to ensure comparability. Model selection was based on 5-fold cross-validation, followed by nested 5-fold cross-validation for the chosen XGBSE StackedWeibull model to obtain an unbiased generalization estimate after feature filtering. No hyperparameter optimization was performed; default configurations were used to isolate the effect of feature engineering and model choice. Retaining the nested structure ensured that each outer fold remained independent of all steps applied in the inner loop.

To test robustness against potential timeline confounding, the top feature (peak_session_freq_count) was residualized with respect to follow-up duration, and all models were re-evaluated. StackedWeibull retained high discrimination (C-index $=0.795$) and consistent calibration, confirming that predictive performance was not solely driven by duration-related effects.

Bootstrap resampling was used to quantify uncertainty in survival estimates, producing 90% confidence intervals without distributional assumptions. Churn labeling used a global cutoff based on duration percentiles to maintain consistent survival contexts across users. While this fixed-window approach avoids endpoint bias and ensures uniform censoring criteria, it assumes that the same inactivity period indicates churn for all user types. Our analysis across three window lengths (30, 60, and 90 days) showed that while the shorter window increased discrimination, there is a higher likelihood it may elevate false positive rates from temporary inactivity. Consistent with “Churn definition and cutoff strategy”, the 60-day window avoided both excessive false positives from shorter windows and minimal improvement from longer windows, while accounting for seasonal patterns.

### Model choice in relation to feature characteristics

Performance patterns in Table 3 illustrate the discrimination–calibration trade-off. CoxPH achieved the highest C-index (0.830) but had the poorest calibration (IBS $=0.106$). RSF offered better calibration (0.094) but lower discrimination. XGBSE KaplanNeighbors achieved low IBS (0.079) but the weakest discrimination among the top models. StackedWeibull balanced these objectives, with strong discrimination (0.826) and the lowest IBS (0.078), and maintained this balance after residualization.

The behavioral features used here are nonlinear, heavy-tailed, and interaction-rich (e.g., std_weekly_sessions, location_switch_rate, session_freq_trend). CoxPH’s proportional hazards and linearity assumptions limit its fit to such data. Tree-based methods such as RSF and gradient boosting in XGBSE impose fewer parametric constraints and can model non-monotonic effects without explicit specification. Stacking gradient boosting with a parametric Weibull AFT model in XGBSE yields calibrated survival probability estimates while retaining expressive modeling power. These findings align with prior work showing hybrid/tree-based survival models often outperform CoxPH in nonlinear or non-proportional hazards settings^25,26^.

### Limitations

Several limitations should be noted. First, the dataset covers Slovenia and nearby countries, potentially limiting generalizability to markets with different adoption rates, infrastructure availability, or policy contexts. Second, only users with at least 30 charging sessions were included, excluding low-frequency users who may exhibit different churn dynamics. This selection may bias results toward habitual users and limit applicability to occasional adopters.

Third, the churn definition used a fixed 60-day inactivity window. This choice may not equally reflect disengagement across all user types (e.g., commuters vs. occasional users). Planned robustness tests with 30- and 90-day thresholds will help quantify the sensitivity of model performance to this choice.

Fourth, demographic and contextual covariates (e.g., income, vehicle type, private charging access) were unavailable and thus omitted, potentially leaving relevant variation unexplained. External time-varying covariates such as weather patterns, holidays, or infrastructure outages were also unavailable. These contextual factors could influence engagement dynamics and provide valuable insights when integrated with behavioral features.

Fifth, although residualization reduced duration dependence in the top feature, some temporal confounding may remain, highlighting the difficulty of fully separating behavioral from timeline effects. 

Finally, while the StackedWeibull model performed well in both calibration and discrimination, it imposes a parametric survival form. Nonparametric, neural, or Bayesian survival models could offer greater flexibility or richer uncertainty estimates in future work.

### Future directions

Future extensions include stratified survival models to capture cohort-level effects, incorporation of time-varying covariates such as weather or weekday patterns, and ensemble approaches combining multiple learners. Deep survival methods could improve modeling of complex temporal dependencies, while Bayesian methods could enhance uncertainty quantification.

From a data perspective, incorporating demographic and contextual attributes, expanding to multiple regions, and explicitly modeling spatial–temporal infrastructure effects (e.g., charging station coverage linked to vehicle availability) could improve generalizability. Linking survival outcomes to infrastructure development and network accessibility may provide actionable insights for service operators and policymakers.

Beyond model architecture improvements, hyperparameter optimization represents a practical next step for deployment. While default settings enabled fair comparison, tailored tuning for tree-based methods like RSF and XGBSE could yield meaningful performance gains. Additionally, validating whether our key behavioral indicators—particularly declining engagement momentum and local station stability – reliably predict churn among underrepresented groups (infrequent users, commercial fleets, new adopters) and across different regional and infrastructural contexts would strengthen the transferability of churn prediction frameworks.

## Supplementary Information

Supplementary Information.

## Data Availability

An anonymized dataset is available from the corresponding author upon reasonable request.

## Abbreviations

C-index: Concordance index
CoxPH: Cox proportional hazards (model)
CV: Cross-validation
DBCE: Debiased binary cross-entropy (model in XGBSE)
EV: Electric vehicle
ICE: Individual conditional expectation
IBS: Integrated Brier Score
KN: KaplanNeighbors (model in XGBSE)
PDP: Partial dependence plot
PHEV: Plug-in hybrid electric vehicle
RFM: Recency-frequency-monetary (user segmentation framework)
RSF: Random survival forests (model)
SHAP: SHapley Additive exPlanations
SW: StackedWeibull (model in XGBSE)
VIF: Variance inflation factor
