# Supplementary material for: Survival analysis of electric vehicle charging behavior and the temporal evolution of feature effects
Source: Sci Rep. 2025 Oct 7;15:34897. doi: 10.1038/s41598-025-18771-8 (PMC12504540; doi:10.1038/s41598-025-18771-8)
Supplement: Supplementary file 1 — Supplementary Information. [file 41598_2025_18771_MOESM1_ESM.pdf]

Supplementary Information for

Survival Analysis of Electric Vehicle Charging Behavior and the Temporal Evolution of Feature Effects

Matej Meža[1,+], Gregor Strle[1,2,+], and Marko Meža[1,*+]

[1]Faculty of Electrical Engineering, University of Ljubljana, Ljubljana, Slovenia
[2]ZRC SAZU, Ljubljana, Slovenia
[*]marko.meza@fe.uni-lj.si
[+]these authors contributed equally to this work

Session Frequency, Inter-Session Gaps, and Churn Window Sensitivity

We examined charging session frequency, inter-session gap distributions, and churn-window sensitivity to determine a robust inactivity threshold for churn labeling.

For the modeling sample (users with ≥ 30 sessions), average usage was 4.6 sessions per active month (once every 6–7 days). Inter-session gaps were skewed: median = 1.0 day, 75th percentile = 5.0 days, and 90th percentile = 13.0 days, indicating that even engaged users occasionally take multi-week breaks (Figure S1).

A 30-day inactivity window is more than twice the 90th percentile gap but is vulnerable to seasonal bias: the global reference date (75th percentile of last-session dates) is 13 December 2024, just before the Christmas/New Year holiday period (Figure S2). Short windows risk misclassifying holiday pauses as churn.

Window-size sensitivity analysis (Figure S3) shows churn prevalence falling from $\sim$40% at 30 days to $\sim$32% at 60 days, with minimal change beyond 90 days. A 60-day window mitigates seasonal mislabeling while maintaining sufficient churn events.

Minimum-session threshold analysis (Figure S4) shows that higher thresholds select longer-tenured, higher-intensity users, but long gaps persist even among the most active.

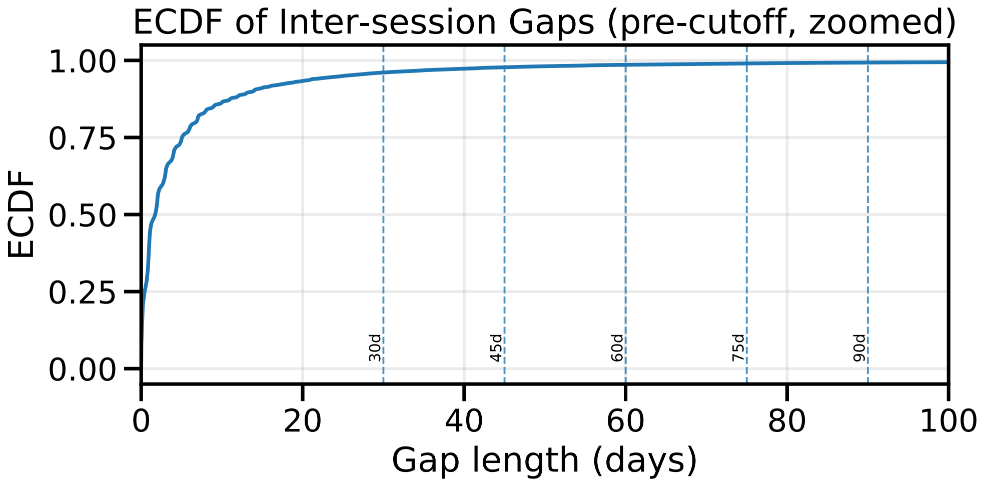

Supplementary Figure 1. **Distribution of inter-session gaps prior to churn cutoff.** ECDF of gaps between consecutive charging sessions for users with ≥ 30 sessions. Vertical dashed lines indicate candidate churn windows (30–90 days). The 90th percentile gap is $\sim$13 days, showing that even highly active users sometimes have breaks of two or more weeks. This distribution provides an empirical baseline for setting inactivity thresholds.

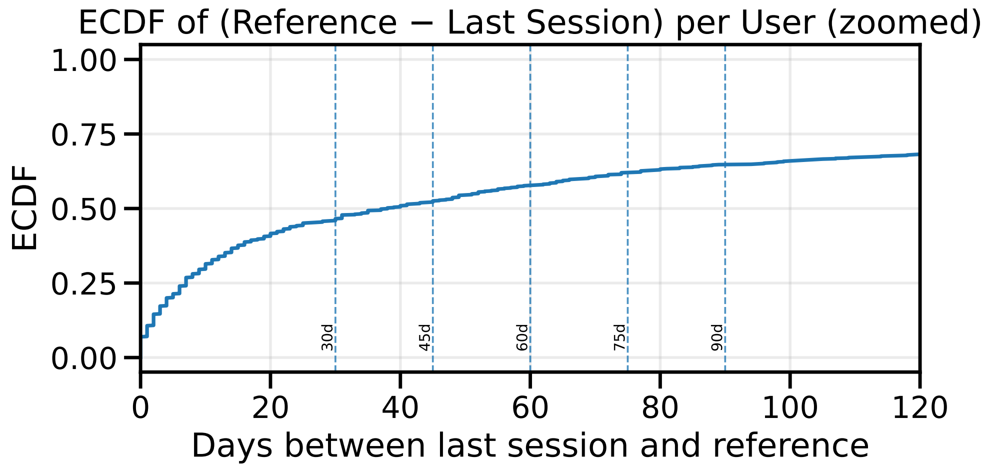

Supplementary Figure 2. **Proximity of last session to the global reference date.** ECDF of the number of days between each user's last recorded session and the global reference date (75th percentile of last-session dates, 13 December 2024). The pronounced clustering near mid-December reflects the seasonal holiday dip in charging activity. Short inactivity windows risk labeling such seasonal pauses as churn.

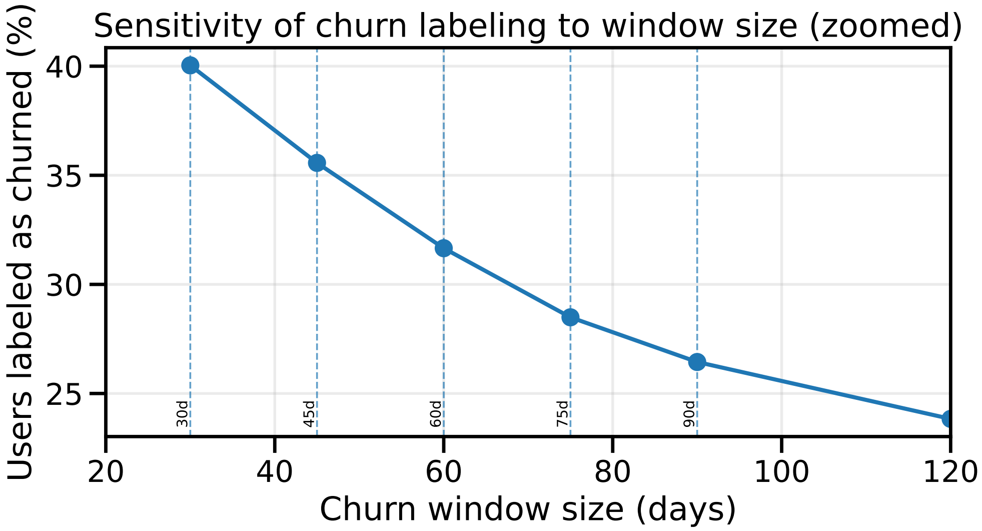

Supplementary Figure 3. **Effect of inactivity window length on churn prevalence.** Churn prevalence declines from ∼40% at a 30-day window to ∼32% at 60 days, with little change beyond 90 days. A 60-day window balances reduced seasonal mislabeling with retention of sufficient churn events for statistical analysis.

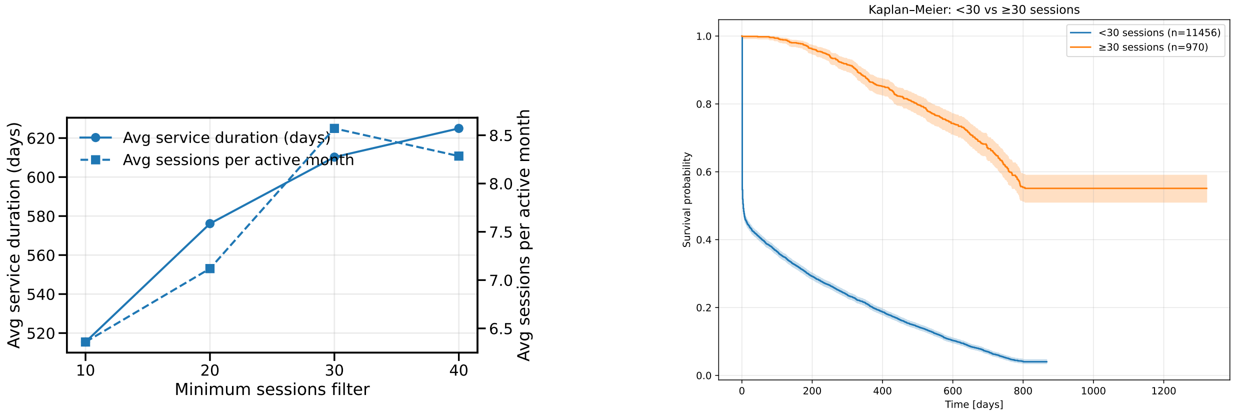

Supplementary Figure 4. **Survival stability and engagement across minimum-session thresholds.** Left: Average service duration (days) and average monthly sessions for thresholds between 10 and 40 sessions. Higher thresholds yield longer tenures and slightly higher monthly activity, but substantial inter-session gaps persist even at the strictest filters. Right: Kaplan–Meier survival curves for ≥ 30 vs. < 30 sessions. The ≥ 30-session group shows markedly higher survival probability (median not reached, indicating that more than half of these users remained active at the end of the observation period) than the < 30-session group (median = 4 days; log–rank $\chi^2 = 1651.2$, $p < 0.001$; Cox HR = 0.138, 95% CI [0.123, 0.154]).

Residual Feature Validation

To test for timeline leakage, we evaluated a StackedWeibull model variant using a residualized `peak_session_freq_count` feature orthogonalized to survival duration via linear regression. This variant showed only a modest drop in performance (nested C-index: 0.797 vs. 0.824), confirming robustness of the original feature.

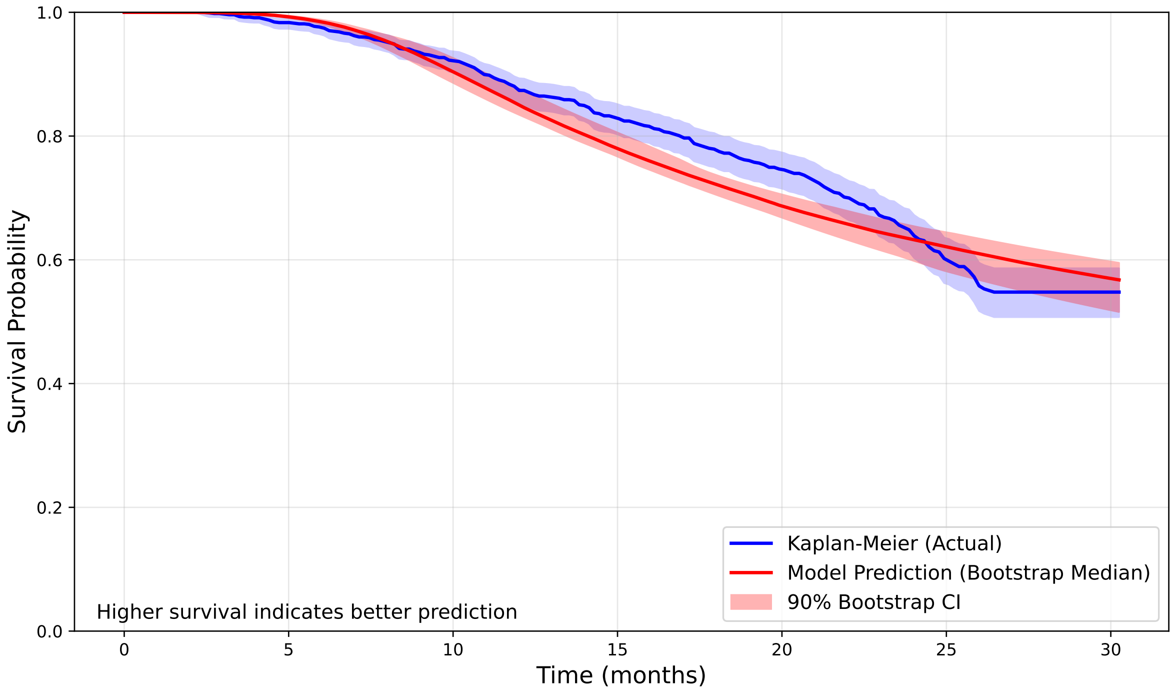

Supplementary Figure 5. **Calibration and uncertainty for the residualized model.** Survival predictions from the StackedWeibull model using a residualized `peak_session_freq_count` feature. The median predicted survival (solid line) closely matches the Kaplan–Meier curve, with 90% bootstrap confidence bands indicating low-to-moderate uncertainty over time.

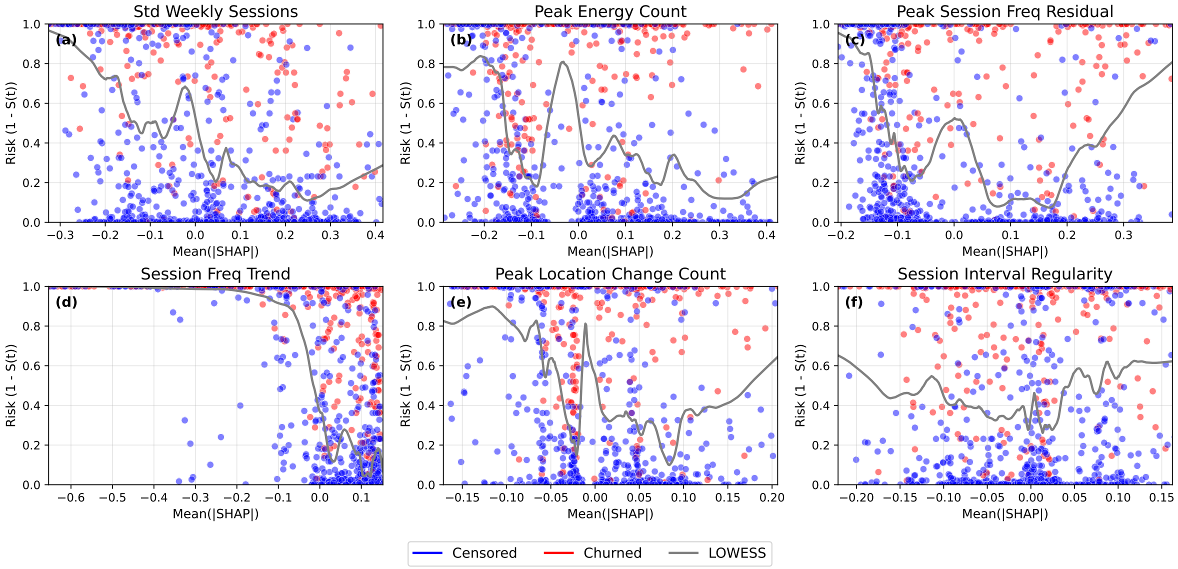

Supplementary Figure 6. **SHAP intensity versus churn risk for residualized features.** Scatterplots of mean absolute SHAP values versus predicted churn risk for six top features in the residualized model. Grey LOWESS curves show average trends. Patterns remain consistent with the original model, indicating stability of feature attributions.

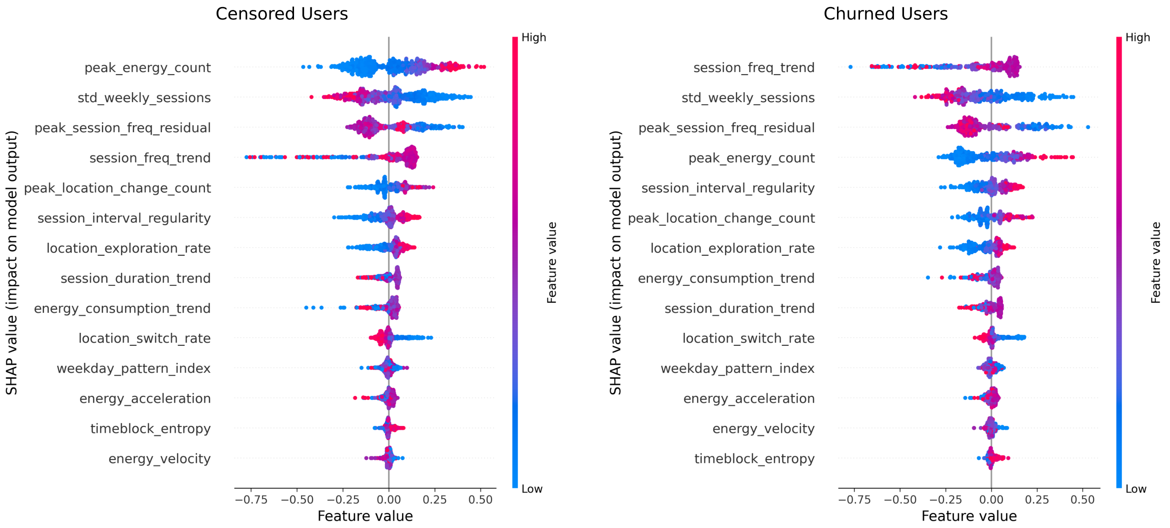

Supplementary Figure 7. **Global feature importance in the residualized model.** SHAP summary plot showing contribution magnitude and direction for each feature. Rankings and effect signs are consistent with the original model, supporting the robustness of predictor relevance.

Temporal SHAP Effects

We assessed how feature effects evolve across time by computing SHAP-based partial dependence curves for all 14 features at three representative survival durations: 11.9, 20.3, and 25.5 months (Figure S8).

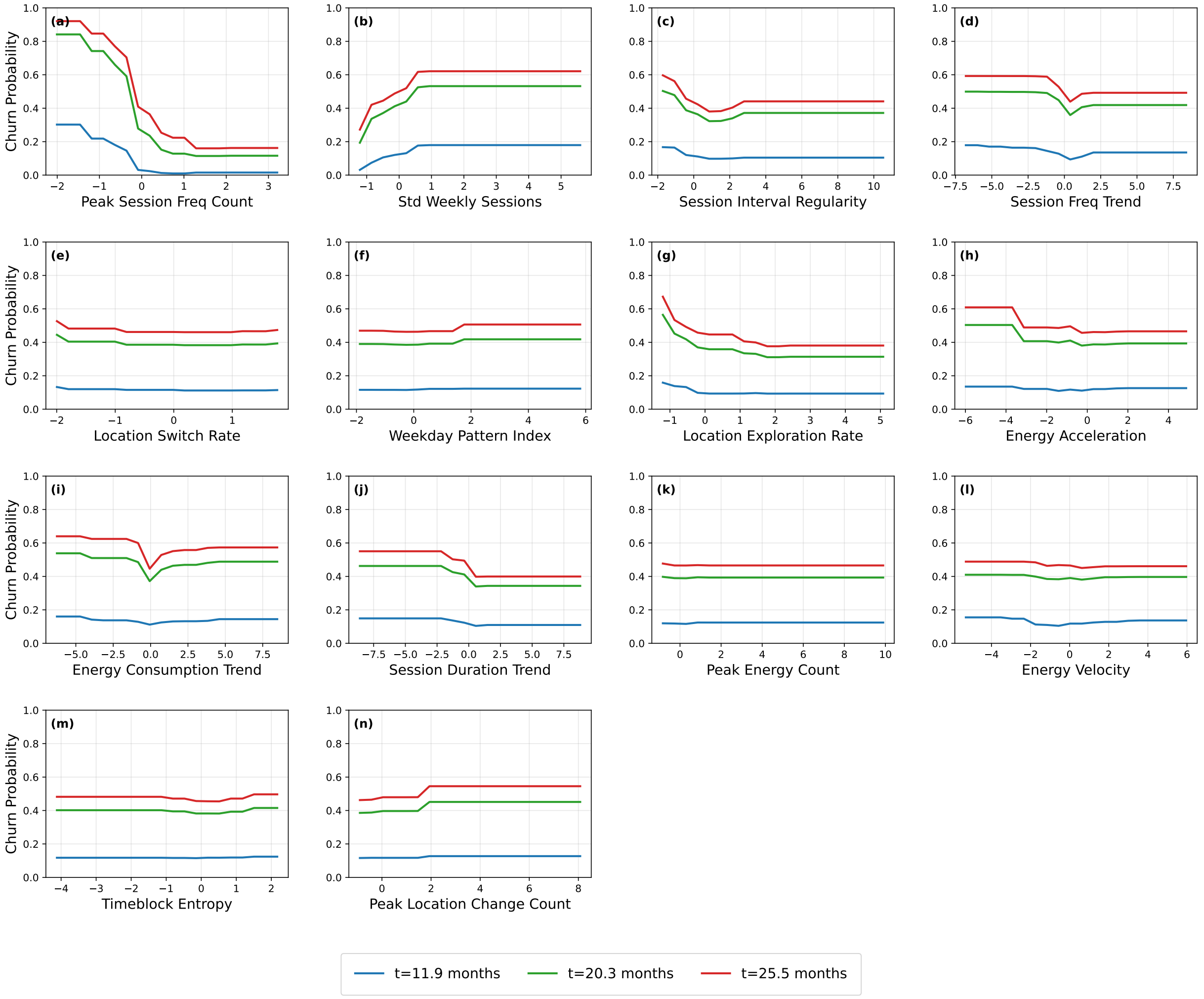

Supplementary Figure 8. **Temporal evolution of feature effects on churn risk.** SHAP partial dependence curves for all 14 features at 11.9 months (blue), 20.3 months (green), and 25.5 months (red). Patterns include amplification of risk separation, inversion of effects (e.g., `session_duration_trend`), U-shaped responses (`std_weekly_sessions`), and consistent trends (`weekday_pattern_index`).

Feature Interaction Visualization

To complement the interaction heatmap in the main text, Figure S9 visualizes the joint effects of `location_switch_rate` and `location_exploration_rate`.

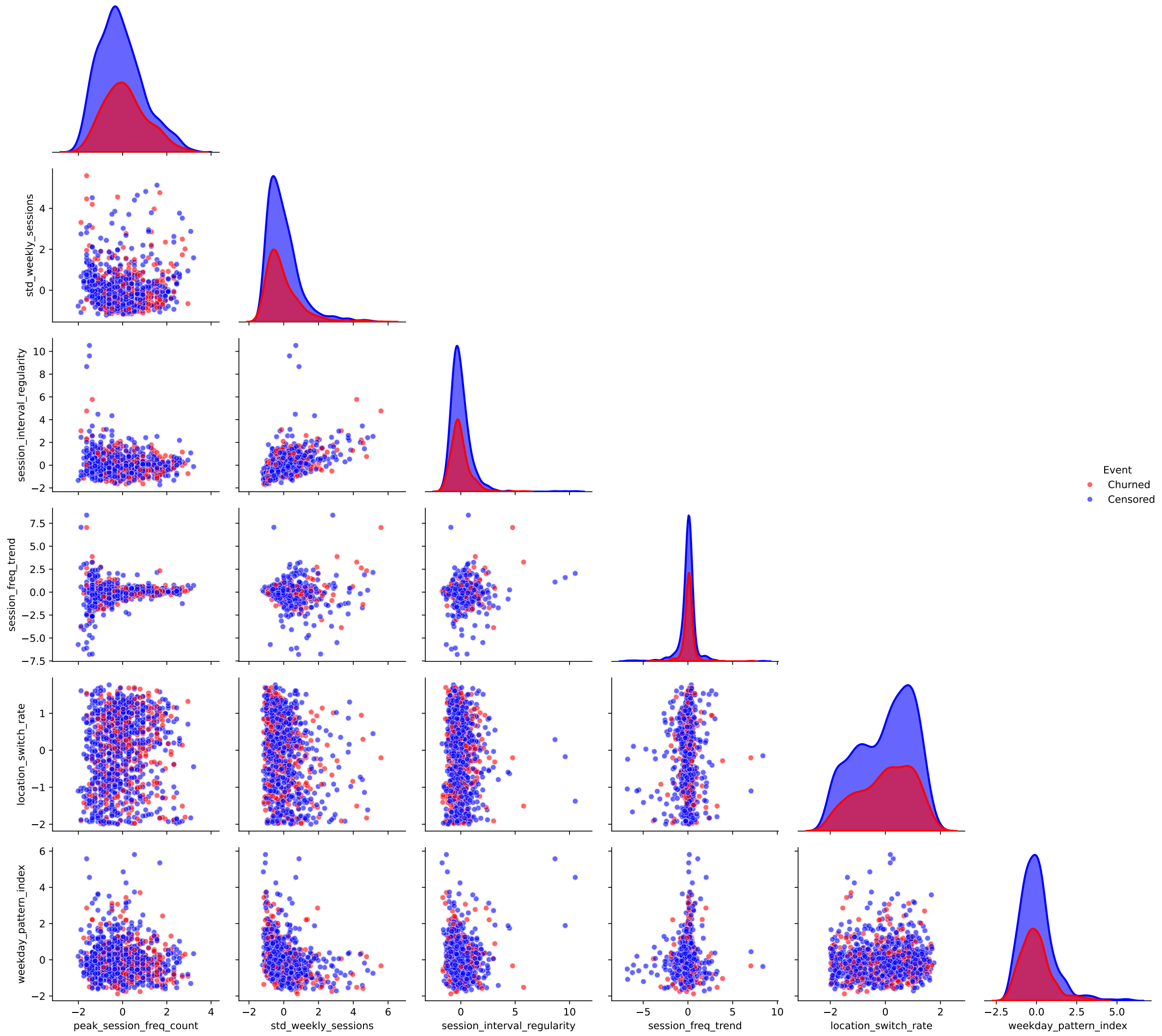

Supplementary Figure 9. **Joint effect of location switching and exploration on churn.** Scatterplot of `location_switch_rate` versus `location_exploration_rate`, colored by survival time. Users with high exploration but irregular switching churn earlier, while frequent switching among a small, familiar set of stations is associated with longer retention, highlighting the protective role of spatial stability.
